# In vivo direct cell-penetrating peptide mediated protein transduction system in *Acyrthosiphon pisum*

**DOI:** 10.1186/s13104-023-06514-9

**Published:** 2023-09-25

**Authors:** Aya Takenaka, Harutomo Konno, Shingo Kikuta

**Affiliations:** College of Agriculture, Ibaraki University, Ami, Inashiki, Ibaraki 300-0393 Japan

**Keywords:** Penetratin, Aphid, Fluorescence, Delivery, Protein transduction

## Abstract

**Objective:**

The principal delivery method for CRISPR-based genome editing in insects is now based on microinjection into single cells or embryos. The direct protein transduction systems cannot be employed in aphids because oogenesis occurs without apparent vitellogenesis. Given the limited timing of injection into the embryonic stage in oviparous aphids, a protein delivery system from the hemolymph to the germline and embryos would be a useful tool for genome editing. This study reports a newly developed direct protein delivery system for aphids using cell-penetrating peptides (CPPs). CPPs are short peptides that translocate across the plasma membrane when bound to cargo proteins.

**Results:**

Penetratin (PEN), a widely conserved CPP among insects, was identified in this study. We used mVenus, a recombinant fluorescent protein, as a visual marker for CPP availability assessments, and fused it with PEN by bacterial protein expression. The mVenus-PEN recombinant proteins were introduced into the hemolymph of adult unwinged *Acyrthosiphon pisum* females using a nanoinjector. Fluorescence emitted by mVenus-PEN was observed in various tissues, such as the gut, trachea, bacteriocytes, and their progeny. This study shows that PEN can deliver exogenously expressed proteins into tissues in vivo, indicating that CPPs are powerful tools for protein transduction.

**Supplementary Information:**

The online version contains supplementary material available at 10.1186/s13104-023-06514-9.

## Introduction

The Clustered Regularly Interspaced Short Palindromic Repeat (CRISPR)-based genome editing system can be applied to various species, including non-model insects, which is crucial to develop novel pest control strategies [1]. To date, single guide ribonucleic acid (sgRNA)–Cas9 (CRISPR associated protein 9) ribonucleoprotein (RNP) complexes have been attempted to be delivered using DNA electroporation targeting the Target of Rapamycin (*Tor*) gene in viviparous aphid ovaries, with no indel mutations detected in the target gene in the offspring of treated individuals [2]. Although the CRISPR/Cas9 system has been tested in aphids, its deployment is still challenging due to the difficulty of injecting into aphid embryos because oogenesis occurs without apparent vitellogenesis. The main delivery method for CRISPR-based genome editing in insects is now based on microinjection of a RNP complex with the sgRNA and Cas9 proteins into single cells or embryos [3–5]. Aphids can switch the reproductive mode between viviparous parthenogenesis and sexual reproduction depending on seasonal conditions. The typical annual life cycle of aphids consists of cyclic parthenogenesis, characterized by a sequence of parthenogenetic generations, typically 10 to 30 generations in common species, followed by a solitary sexual generation [6, 7]. Therefore, the aphid life cycle presents challenges for the development of CRISPR-based genome editing due to two distinctive factors: the alternation of reproductive modes and the obligatory diapause experienced by fertilized eggs [8]. Given the limited timing of injection into the early embryo stage in oviparous aphids, a direct protein delivery system from the hemolymph to the germline and early embryos would be a useful tool for genome editing. A protein delivery system using cell-penetrating peptides (CPPs) is available for Cas9 transduction for human cell lines such as embryonic stem cells, dermal fibroblasts, HEK293T cells, HeLa cells, and embryonic carcinoma cells [9]. In this study, we present a novel direct protein delivery system using CPPs in aphids.

CPPs are short peptides enriched in arginine, typically consisting of 4–40 amino acids, that facilitate cell translocation through various mechanisms. These mechanisms can include endocytosis and/or intracellular effects that the peptides themselves promote, or those imparted by cargo delivered either covalently or non-covalently [10, 11]. To date, many CPPs have been identified in naturally derived proteins or artificially designed ones [11, 12]. The precise transduction mechanism mediated by CPPs remains unknown and appears to be both endocytic and non-endocytic, depending on the CPP, cell type, and cargo characteristics [13]. In insects, CPP-mediated protein transduction has been successfully demonstrated in the midgut and cultured cells of lepidopterans, serving as potential enhancers for insecticide delivery [12]. However, it is unknown whether CPP can deliver proteins of interest to aphid tissues. In this study, we aimed to investigate CPP capabilities of transducing exogenous proteins into aphid tissues in vivo.

## Materials and methods

### Insect

*Acyrthosiphon pisum* (pea aphid) unwinged females were reared according to a previous study [14]. Briefly, the aphids were reared on a broad bean (*Vicia faba*) sprout under a 16 L-8D photoperiod at 20 ± 1 °C. All analyses were performed using well-developed adults from six independently prepared lineages to validate reproducibility.

### mVenus-penetratin construction

Penetratin (PEN) peptides, known as CPP, were introduced into the bacterial expression vector mVenus_pRSETB via site-directed mutagenesis following the manufacturer’s instructions (Agilent Technologies, Santa Clara, CA, USA). The primers used were as follows: Forward, 5′-AAATTTGGTTTCAGAACCGCCGCAACAAATGGAAAAAATAGAAGCTTGA-3′ and Reverse, 5′-ATTTTAATCTGGCGCTTGTACAGCTCGTCCATGCCGAG-3′. PCR was performed using the following protocol: 30 cycles of denaturation at 95 °C for 20 s, annealing at 60 °C for 5 s, and extension at 72 °C for 20 s using KOD One PCR polymerase (TOYOBO, Osaka, Japan). mVenus-PEN sequences were confirmed via DNA sequencing (Eurofins Genomics, Tokyo, Japan). Sequence data were analyzed using FinchTV sequence scanner software and aligned using CLC Sequence Viewer 8 (Qiagen, Venlo, Netherlands). The original expression vector mVenus_pRSETB was used as mVenus without CPPs.

### Purification of the recombinant mVenus-PEN

The expression vectors mVenus-PEN_pRSETB and mVenus_pRSETB were transformed into BL21(DE3) cells (Nippon Gene, Tokyo, Japan), respectively. A single colony of the transformant was cultured in 50 mL LB medium supplemented with 50 µg/mL ampicillin for 16 h at 37 °C and then induced to express recombinant mVenus-PEN and mVenus in the presence of 1 mM isopropyl β-d-1-thiogalactopyranoside (IPTG) at 15 °C for 6–12 h. The cells were harvested with centrifugation at 5000 × *g* for 5 min and resuspended in phosphate-buffered saline (PBS). *Escherichia coli* was disrupted by ultrasonication on ice. mVenus-PEN and mVenus were purified using affinity chromatography with Profinity IMAC Ni-charged resin according to the manufacturer’s instructions (Bio-Rad, Hercules, CA, USA). Binding to the resin was performed in batches at 4 °C for 6 h, followed by washing with sterilized PBS and washing thrice with 20 mM Tris-HCl (pH 8.0) containing 10 mM imidazole. Elution was performed using 500 mM imidazole and 20 mM Tris-HCl (pH 8.0). The samples were then stored on ice. Protein concentrations were determined using a Qubit 4 Fluorometer (Thermo Fisher Scientific, Carlsbad, CA, USA). An aliquot of the sensor was heated at 95 °C for 5 min to deactivate it, as the sensor protein emits fluorescence. The recombinant fluorescent proteins were placed at 4 °C in darkness until use. All the analyses were performed using colonies containing independent protein preparations.

### Microinjection

Adult *A*. *pisum* were chilled at − 20 °C for 10 s. The chilled aphids were placed on a 1.5% agar cube and their heads were embedded in agar. The recombinant fluorescent proteins were diluted to 820 ng/µL with PBS. A protein solution (285 nL) was injected into the cauda using NANOJECT II (Drummond Scientific, Broomall, PA, USA). The aphids were incubated at 15 ± 1 °C for 24 h after injections. mVenus fluorescence was observed under a microscope.

### Fluorescence detection in *A. pisum*

The fluorescence of mVenus was observed using a BZ-8100 fluorescence microscope (Keyence, Tokyo, Japan) and an Apexview APX100 all-in-one microscope (Olympus, Osaka, Japan) equipped with an excitation filter of 470/40 nm and emission filter of 535/50 nm. Data acquisition was set at 500–1000 ms at 20 ± 1 °C.

## Results and discussion

We first investigated the efficiency of protein delivery to *A*. *pisum* using the transactivating transcription factor (TAT) peptides. TAT, derived from the *Tat* encoded by human immunodeficiency virus (HIV)-1 [15], has been shown to efficiently deliver fluorescent proteins to human embryonic retinoblasts and in vitro cultured columnar cells from the larval midgut of *Bombyx mori* [16], suggesting that TAT peptides may be able to translocate the fused cargo proteins. Fluorescent proteins have been used as visual markers for CPP availability assessments [16, 17]. TAT genetically fused to the C-terminus of mVenus (to avoid the influence of its fluorescence) was obtained using bacterial expression. The purity of the recombinant proteins was examined using SDS-PAGE (Fig. S1). The fluorescence emitted by mVenus-TAT was unclear in *A*. *pisum* even 24 h after injection (Fig. S2), indicating that the mVenus remained in the hemolymph. TAT did not deliver mVenus to the aphid tissue. The TAT peptide is derived from the HIV-1 virus. Given the absence of viral genome expression in insect cultured cells [18], HIV-1 is unsuitable for infecting insects. The efficacy of TAT-mediated protein delivery to insects, including *A*. *pisum*, may be limited.

**Fig. 1:**
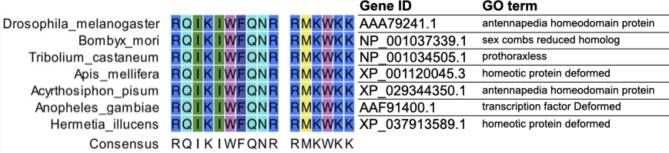
Conservation of cell-penetrating peptides (CPPs) in insects. Derived CPP sequences were obtained from penetratin (PEN), known as the homeodomain of Antennapedia, derived from *Drosophila melanogaster*. CPP candidates equivalent to PEN in several insects were obtained from the BLAST protein database in NCBI. The alignment of CPPs, NCBI IDs and the Gene Ontology (GO) terms is shown for several insects

Next, we investigated insect-derived CPP candidates to replace the TAT peptide. PEN, a 16-residue peptide from the homeodomain of *Antennapedia* (a *Drosophila* homeoprotein) can take up fused proteins via energy-independent endocytosis [17, 19, 20], and deliver cargo to rat mesencephalic cells [17]. The *D*. *melanogaster*-derived PEN has the potential to transport proteins into aphid tissues, however, the transport efficiency would be affected by the amino acid residues constituted of PEN. Therefore, we hypothesized that a PEN endogenous in aphids or widely conserved among insect species would be a good candidate as CPP. Homologous *Antennapedia* genes were predicted using a protein BLAST search in the NCBI database using the *D*. *melanogaster* PEN sequences as a query to obtain the intrinsic features for *A*. *pisum*. PEN sequences were abundant not only in Diptera (*Anopheles gambiae* and *Hermetia illucens*), but also in other insects such as *Bombyx mori*, *Tribolium castaneum*, *Apis mellifera*, and *A*. *pisum* (Fig. 1). The PEN sequence was identical among these insects, indicating that PEN can deliver cargo to the tissues of *A*. *pisum*. Next, we tested the delivery efficacy of the mVenus fused PEN by microinjecting them into the hemolymph of *A*. *pisum*.

**Fig. 2:**
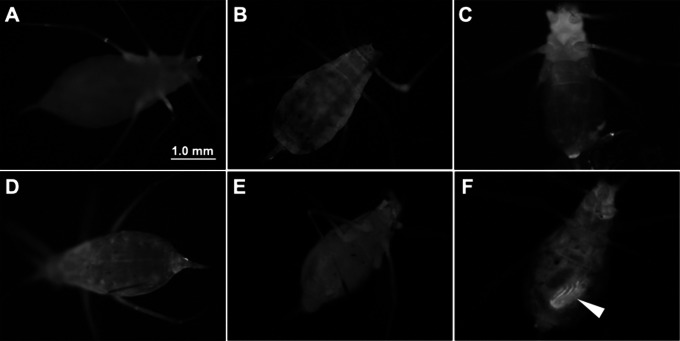
Cell-penetrating peptides (CPPs) are essential for the delivery of cargo proteins to the tissues. **(A)** Non-injected control aphid. **(B)** Phosphate-buffered saline (PBS)-injected aphids. **C and D**. mVenus fluorescence was observed 30 min after injection. mVenus-without-CPP-injected aphids **(C)**; mVenus-PEN injected aphids **(D)**. **E and F**. The mVenus fluorescence was observed 24 h after injection. mVenus without penetratin (PEN)-injected aphids **(E)**; mVenus-PEN-injected aphids **(F)**. Arrowhead represents an embryo

Adult *A*. *pisum* that were not injected or sham-injected with PBS showed slight autofluorescence (Fig. 2A, B). Neither mVenus-PEN nor mVenus showed fluorescence 30 min after injection (Fig. 2C, D). Cultured columnar cells of *B*. *mori* showed low fluorescence signals after 30 min of incubation, which then increased for 24 h [16]. In this study, mVenus-PEN fluorescence was observed 24 h after injection, but no signals were observed in any tissue using mVenus without PEN (Fig. 2E, F). Fluorescence was observed in the mature embryos (Fig. 2F). These results indicate that PEN-mediated protein delivery to tissues in the open-circulating hemolymph of insects occurred within 24 h.

**Fig. 3:**
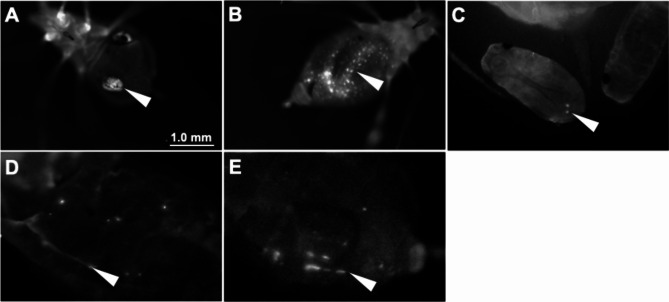
Transduction of mVenus-PEN into *Acyrthosiphon pisum*. The mVenus fluorescence of tissues in *A*. *pisum* was observed 24 h after injection. **(A)** Bacteriocyte. **(B)** Gut. **(C)** Bacteriocyte in embryo. **(D)** Medial nerve. **(E)** Trachea

Protein delivery into the embryo was successful in parthenogenetic viviparous female aphids when we injected mVenus-PEN solution into the cauda. We attempted to inject mVenus-PEN between the internodes of the head and thorax, thorax and abdomen, and abdomen and cornicles of adult aphids; however, most of the aphids died within 24 h. Microinjection into the cauda of adult *A*. *pisum* resulted in a high survival rate, regardless of the presence or absence of PEN (Fig. S3). There are no significant differences in aphid survival from control PBS injections, indicating that PEN is not toxic at all (Fig. S3). An increase in the amphipathicity of PEN through mutations and genetic engineering increases the toxicity of the peptide, rather than its transduction efficiency [20]. Accordingly, the examination of toxicity in aphids by using a novel CPP is important, as is the protein delivery efficiency. The safe release of mVenus-PEN into the aphid hemolymph requires careful physical manipulation of the injection capillaries. Under the stereomicroscope, gently insert the injection capillary into the cauda of the aphid, allowing it to approach the embryo slowly. The accumulation of arginine-rich peptides such as TAT and PEN on the cell surface can facilitate the transport of cargo proteins into the cell [21]. Microinjection of mVenus-PEN into the near target tissues allowed uptake into the bacteriocytes (Fig. 3A), gut (Fig. 3B), bacteriocytes in embryos (Fig. 3C; Supplemental video), medial nerve (Fig. 3D), and trachea (Fig. 3E). Despite this, the success rate of exogenous protein transduction into these tissues remained at most 33% (n = 114). In the insect cultured cell line Sf9, CPPs derived from a mammalian protein and artificial peptides facilitated uptake into the cells than penetratin [22], indicating that the protein delivery system via CPPs is diversified in insects. Elucidating the mechanism of protein delivery using CPPs could potentially lead to increased implementation efficiency.

To date, several technologies have been developed to achieve the Cas9-mediated gene editing in insects. Cas9 was delivered into developing mosquito oocytes using proline-to-cysteine substitution peptides consisting of 41 amino acids derived from *D*. *melanogaster* Yolk protein 1 (DmYP1) from the female hemolymph [23]. A newly developed Cas9 RNP delivery system *via* the endocytosis of vitellogenic oocytes from the hemolymph during vitellogenesis in *Blattella germanica* and *Tribolium castaneum* has successfully produced gene-edited offspring [24]. However, the direct protein transduction systems for the purpose of the CRISPR-based genome editing cannot be employed in aphids because oogenesis occurs without apparent vitellogenesis. The CPP can be an aid in the delivery of proteins such as Cas9 to the aphid tissue.

The direct protein transduction systems using CPPs also hold promise for exploring molecular features of aphid physiology. For example, bacteriocytes harboring the endosymbiont *Buchnera* in *A. pisum* take up glutamine and trehalose via proton gradient transporters expressed in the plasma membranes [14, 25]. Although these findings provide biochemical insights, such as expression in *Xenopus* oocytes, functional analysis is required to establish their physiological relevance. To validate the physiological mechanisms underlying these processes, genetically encoded sensors, such as FLIPQ-TV3.0 [26] and Tre-C04 [27], are available for the detection of glutamine and trehalose in bacteriocytes. Addressing these questions by fusing CPP with protein-based sensors instead of using mVenus would provide valuable insights.

### Limitations

Further studies are needed to elucidate the mechanism of protein delivery using CPPs and increase the efficiency of implementation. Understanding the protein delivery mechanisms in aphids and various insects is important for the convenient and efficient use of CPP-mediated protein delivery systems. In addition, a protein delivery system using CPPs can potentially be applied for Cas9 transduction. Therefore, future studies in this area are important for developing effective genetic engineering techniques for insects.

### Electronic supplementary material

Below is the link to the electronic supplementary material.

Supplemental Video

Supplemental Figure 1

Supplemental Figure 2

Supplemental Figure 3

Supplemental Notes

## Data Availability

Data will be made available on request.

## Abbreviations

CPP: Cell-penetrating peptide
CRISPR: Clustered Regularly Interspaced Short Palindromic Repeat
PBS: Phosphate-buffered saline
TAT: Transactivating transcription factor
PEN: Penetratin
RNP: Ribonucleoprotein
