# Supplementary figures and images for: In vivo direct cell-penetrating peptide mediated protein transduction system in Acyrthosiphon pisum
Source: BMC Res Notes. 2023 Sep 25;16:231. doi: 10.1186/s13104-023-06514-9 (PMC10521536; doi:10.1186/s13104-023-06514-9)

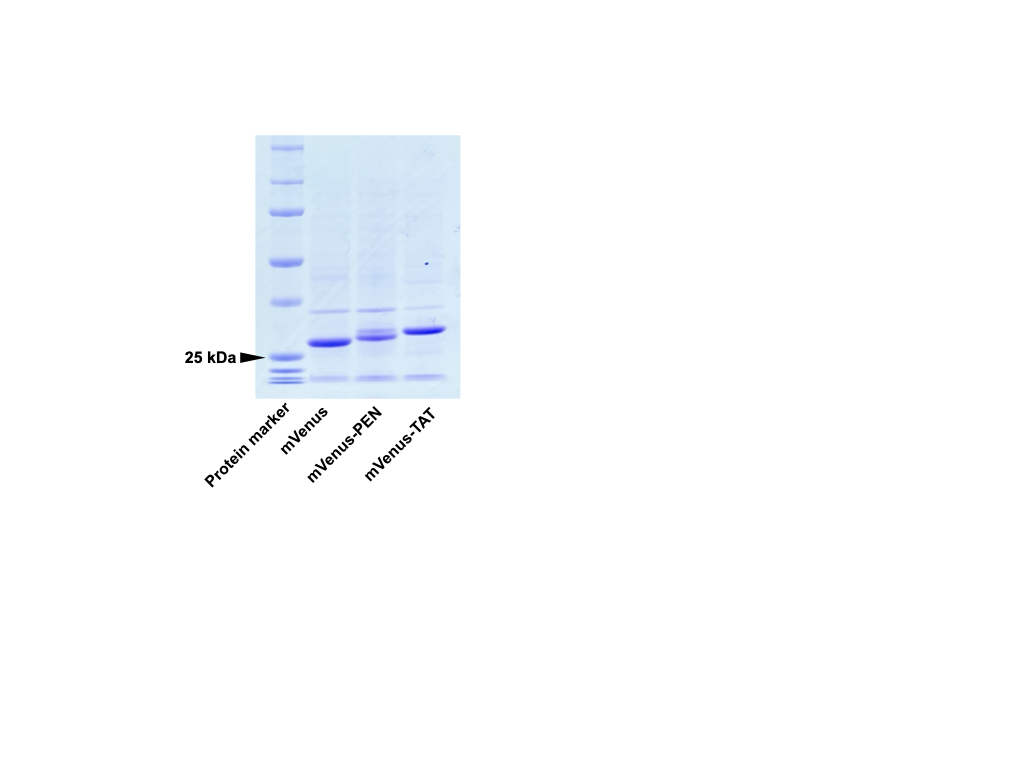

Supplement: Supplementary file 2 — Supplemental Figure 1 [file 13104_2023_6514_MOESM2_ESM.tiff]

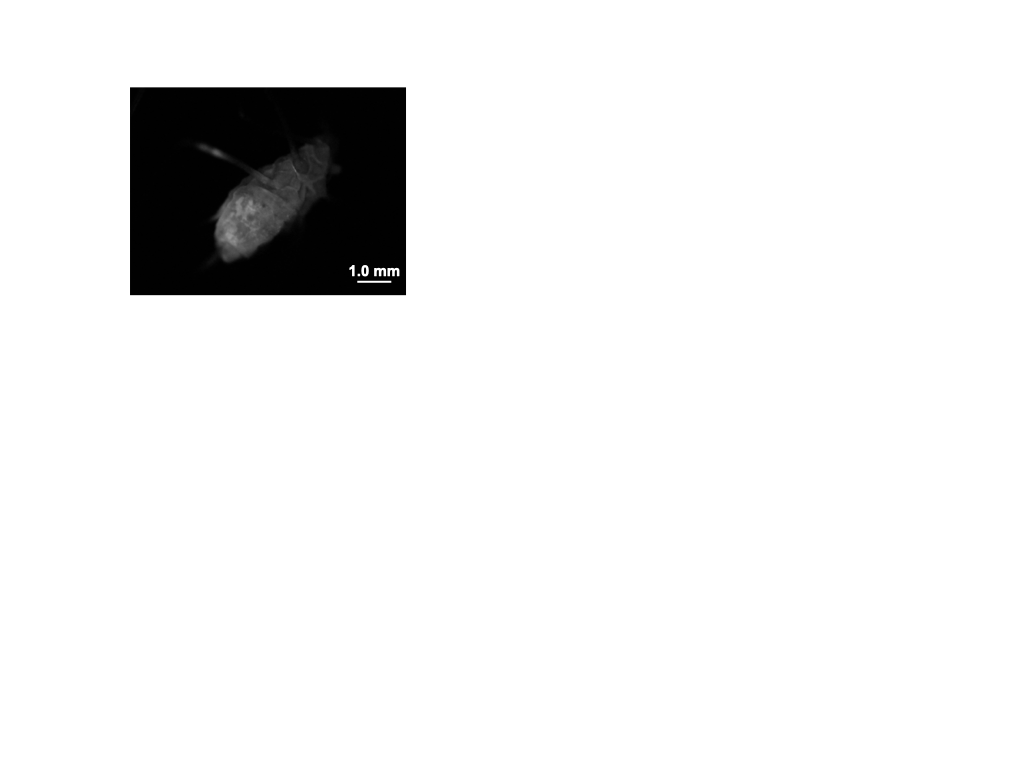

Supplement: Supplementary file 3 — Supplemental Figure 2 [file 13104_2023_6514_MOESM3_ESM.tiff]

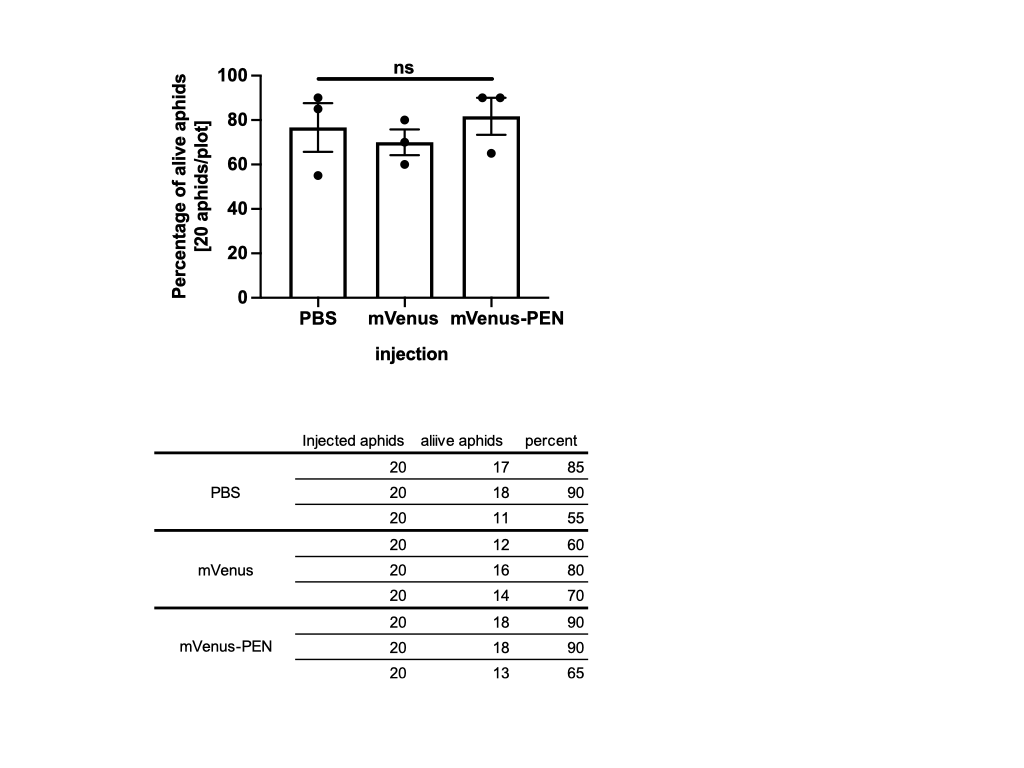

Supplement: Supplementary file 4 — Supplemental Figure 3 [file 13104_2023_6514_MOESM4_ESM.tiff]
