# Supplementary material for: In vivo direct cell-penetrating peptide mediated protein transduction system in Acyrthosiphon pisum
Source: BMC Res Notes. 2023 Sep 25;16:231. doi: 10.1186/s13104-023-06514-9 (PMC10521536; doi:10.1186/s13104-023-06514-9)
Supplement: Supplementary file 5 — Supplemental Notes [file 13104_2023_6514_MOESM5_ESM.docx]

**Supplemental figures**

**Fig. S1. The purity of the recombinant proteins.**

The purity of the recombinant proteins was analyzed using SDS-PAGE. The protein maker (Precision Plus Protein™, Bio-Rad) is used to estimate molecular weights.

**Fig. S2. Transduction of mVenus-TAT into *Acyrthosiphon pisum***

Fluorescence was emitted by mVenus-TAT after 24 h of injection. The construction of mVenus-TAT is described in the supplemental notes. Experimental methods for protein purification and microinjection were examined using mVenus-PEN. Representative images are shown in this section.

**Fig. S3. Toxicity of mVenus-PEN against wingless adult *Acyrthosiphon pisum***

Error bars represent standard error (n = 3). Each plot represents the percentage survival of 20 aphids at 24 h after injection. Toxicity assays were repeated at least thrice as biological replicates and representative data are shown. Statistical analyses were performed using Prism 8 software (GraphPad Software, San Diego, CA, USA). Statistical significance was determined using one-way ANOVA (*p* = 0.6504). The raw data are shown.

**Supplemental notes**

**mVenus-TAT construction**

The TAT peptides (TAT) were introduced into the bacterial expression vector mVenus_pRSETB by using the site-directed mutagenesis. PCR reactions and PCR polymerase were described in the materials and methods. Primers used were as follows: Forward, 5'-TGTACAAGTATGGCCGCAAAAAACGCCGCCAGCGCCGCCGCTAACTCGAGAAGCTTGATCC-3' and Reverse, 5'-CGGCCATACTTGTACAGCTCGTCCATG-3'. The mVenus-TAT sequences were confirmed by DNA sequencing (Eurofins Genomics, Tokyo, Japan). Sequence data were analyzed using the FinchTV sequence scanner software and were aligned using the CLC Sequence Viewer 8 (Qiagen).

**Purification of the recombinant mVenus-TAT**

The recombinant mVenus-TAT was obtained from the bacterial expression as described in "Purification of the recombinant mVenus-PEN" in the materials and methods. The purity of the recombinant proteins was verified using SDS-PAGE. The electrophoresis was performed under 100V and 20 mA. The gel was stained using Coomassie blue.

**Supplemental video**

**The mVenus-PEN uptake to the bacteriocytes in the embryo of *A*. *pisum*.**

Arrowhead represents the inset.
